# Roles for Sleep in Neural and Behavioral Plasticity: Reviewing Variation in the Consequences of Sleep Loss

**DOI:** 10.3389/fnbeh.2021.777799

**Published:** 2022-01-20

**Authors:** Jacqueline T. Weiss, Jeffrey M. Donlea

**Affiliations:** ^1^Department of Neurobiology, David Geffen School of Medicine at University of California, Los Angeles, Los Angeles, CA, United States; ^2^Neuroscience Interdepartmental Program, University of California, Los Angeles, Los Angeles, CA, United States

**Keywords:** sleep, plasticity, review, memory, *Drosophila*

## Abstract

Sleep is a vital physiological state that has been broadly conserved across the evolution of animal species. While the precise functions of sleep remain poorly understood, a large body of research has examined the negative consequences of sleep loss on neural and behavioral plasticity. While sleep disruption generally results in degraded neural plasticity and cognitive function, the impact of sleep loss can vary widely with age, between individuals, and across physiological contexts. Additionally, several recent studies indicate that sleep loss differentially impacts distinct neuronal populations within memory-encoding circuitry. These findings indicate that the negative consequences of sleep loss are not universally shared, and that identifying conditions that influence the resilience of an organism (or neuron type) to sleep loss might open future opportunities to examine sleep's core functions in the brain. Here, we discuss the functional roles for sleep in adaptive plasticity and review factors that can contribute to individual variations in sleep behavior and responses to sleep loss.

## Introduction

Sleep is a physiological state that has been conserved across evolution, even noted in invertebrates lacking a centralized brain (Hendricks et al., 2000; Shaw et al., 2000; Zhdanova et al., 2001; Raizen et al., 2008; Singh et al., 2014; Nath et al., 2017). Although sleep's physiological functions remain poorly understood, sleep loss has been associated with deleterious effects on health and cognition (Rechtschaffen and Bergmann, 1995; Dinges et al., 1997; Durmer and Dinges, 2005; Spiegel et al., 2005; Banks and Dinges, 2007; Knutson et al., 2007; Grandner et al., 2010). Sleep varies based on previous waking experience (Ganguly-Fitzgerald et al., 2006; Huber et al., 2007; Hanlon et al., 2009; Keene et al., 2010; Beckwith et al., 2017; Kirszenblat et al., 2019; Milinski et al., 2021) throughout the lifespan (Roffwarg et al., 1966; Kales et al., 1967; Feinberg and Carlson, 1968; Cauter et al., 2000; Backhaus et al., 2007; Dijk et al., 2010; Feinberg and Campbell, 2010; Carrier et al., 2011; Vienne et al., 2016; Mander et al., 2017), and between species (Lyamin et al., 2008, 2017, 2018; Siegel, 2008; Lesku et al., 2012), suggesting that sleep has multiple functions. However, because sleep coincides with broad changes in neurophysiology and necessitates a loss of consciousness with reduced responsiveness to external threats, it is likely that sleep evolved, at least in part, to support brain function (Rasch and Born, 2013; Tononi and Cirelli, 2014). Notably, sleep is often elevated during periods of synaptic reorganization, including early development (Roffwarg et al., 1966; Shaw et al., 2000; Kayser et al., 2014), recovery from neural injury (Singh and Donlea, 2020; Stanhope et al., 2020), and memory consolidation (Walker et al., 2002; Ganguly-Fitzgerald et al., 2006). These findings each suggest that sleep supports plastic remodeling in the brain. Synaptic plasticity allows behavioral flexibility in response to external stimuli, and enables the processing and storage of information (Hughes, 1958; Zucker and Regehr, 2002; Cooke and Bliss, 2006). However, the underlying cellular and molecular mechanisms that support plasticity during sleep remain an area of intense investigation.

The impacts of sleep loss, interestingly, vary widely depending on age, environmental conditions, and genotype. While organisms typically recover from acute sleep disruptions relatively quickly, early-life sleep disruptions can prevent developmental plasticity during critical periods and result in long-lasting changes in circuit connectivity and behavior (Frank et al., 2001; Seugnet et al., 2011; Kayser et al., 2014). Conversely, some individuals withstand sleep loss with few consequences depending on the physiological conditions or genetic factors (Viola et al., 2007, 2012; Lyamin et al., 2008; Keene et al., 2010; Thimgan et al., 2010; Donlea et al., 2012; Lesku et al., 2012). In some cases, sleep disruption even provides an opportunity to weaken maladaptive memories (Poe, 2017). Examining the variables that can influence an individual's sensitivity to sleep loss could provide new insights into the core mechanisms of sleep-dependent plasticity. In this review, we will discuss roles for sleep in the maintenance of neural and behavioral plasticity during development, and learning/memory. Finally, we outline ethologically relevant conditions in which organisms can maintain neural and behavioral plasticity in the face of sleep loss.

## Development

Synaptic plasticity plays a crucial role in brain development, especially in the refining of neural connectivity through the process of pruning (Paolicelli et al., 2011). Defects in synaptic pruning during development are thought to contribute to atypical circuit function seen in neurodevelopmental disorders (Paolicelli et al., 2011; Konopaske et al., 2014; Tang et al., 2014; Cossío et al., 2017; Kim et al., 2017; Neniskyte and Gross, 2017). Daily sleep amounts peak in many species early in development, when the brain is undergoing significant plastic changes (Roffwarg et al., 1966; Jouvet-Mounier et al., 1970; Shaw et al., 2000; Kayser et al., 2014). Studies in humans have found that sleep disruption during development is associated with severe and lasting consequences for behavior and cognition (O'Brien et al., 2004; Halbower et al., 2006; Ednick et al., 2009). While these human studies provide a correlational link between impaired sleep and later cognition, several lines of animal studies described below indicate conserved roles for sleep in neurodevelopment of several species and begin to identify possible mechanisms by which sleep might influence brain development.

Rapid eye movement (REM) sleep is thought to play a particularly important role in development. Infants spend as much as 50% of their time asleep in REM, compared to 25% in adults (Roffwarg et al., 1966; Jouvet-Mounier et al., 1970). This period of increased REM sleep coincides with heightened formation and elimination of synapses in the developing mouse brain (Marks et al., 1995). Previous work found that REM deprivation, but not non-REM (NREM) deprivation, prevents the elimination of newly-formed dendritic spines in layer V pyramidal neurons in the developing mouse motor cortex (Li et al., 2017). Further, elimination of recent spines during REM facilitates the development of new spines at nearby sites. While most newly formed spines are eliminated, persistent spines are strengthened by REM sleep. Notably, similar findings were observed in the adult mouse brain following motor learning (Li et al., 2017).

A unique feature of REM sleep is the occurrence of myoclonic twitches, or spontaneous, discrete, spastic movements of the limbs (Tiriac et al., 2012; Blumberg et al., 2013; Sokoloff et al., 2020). These twitches occur throughout the mammalian lifespan, but are particularly abundant in infancy (Tiriac et al., 2012; Blumberg et al., 2013; Sokoloff et al., 2020, 2021). The development of myoclonic twitches depends on sensory feedback; the spatiotemporal organization of twitches is disrupted in newborn ErbB2 muscle-specific knockout mice which lack muscle spindles and exhibit impaired proprioception in adulthood (Blumberg et al., 2015). Muscle spindles are sensory receptors that relay changes in the length of muscles to the central nervous system and are necessary for intact proprioception (Kröger and Watkins, 2021). These findings suggest that twitches during sleep provide the developing brain with opportunities to refine immature sensorimotor maps and better coordinate limb movements. Twitching during early-life REM episodes, therefore, could facilitate the transformation of uncoordinated movements during infancy to the fine-tuned sensorimotor maps of an adult. Sensory feedback from twitching limbs are thought to contribute to motor learning and sensorimotor integration (Blumberg et al., 2013, 2020; Sokoloff et al., 2015; Rio-Bermudez and Blumberg, 2018; Glanz et al., 2021), as reafference from myoclonic twitches selectively activates brain regions such as the thalamus, cortex, hippocampus, and cerebellum in infant rats (Khazipov et al., 2004; Mohns and Blumberg, 2010; Tiriac et al., 2012; Sokoloff et al., 2015). Because reafference signals from self-movement are gated during waking, sleep disruptions that interfere with twitching, and their corresponding neuronal activity may disrupt sensorimotor maturation (Tiriac and Blumberg, 2016). While these studies provide an important and promising link between early-life sleep episodes and the development of mature sensorimotor representations, the underlying synaptic mechanisms and long-term consequences of myoclonic twitch disruptions remain to be characterized in detail.

A vital role for sleep in early life plasticity is shared across sensory circuits. The study of ocular dominance plasticity (ODP) induced by monocular deprivation (MD) in cats, for example, is a canonical model of critical period plasticity during development that is reliant upon sleep. During an early critical period for visual development, occluding one eye leads to enhanced visual cortex responses to inputs from the non-deprived eye (Hubel and Wiesel, 1970). Sleep enhances ODP; NREM sleep deprivation prevents enhancement of cortical plasticity, suggesting that sleep is vital for consolidating experience-dependent changes in ocular dominance following MD (Frank et al., 2001). More recent work has found that REM deprivation disrupts cortical plasticity after MD as well, perhaps by disrupting replay-like patterns of activity in the visual cortex (Bridi et al., 2015). Additionally, REM sleep following MD is sufficient to prevent reversal of ODP following subsequent manipulations such as further SD (Bridi et al., 2015), cortical inactivation (Jha et al., 2005), and inhibition of NMDA receptors (Aton et al., 2009). The dependence of ODP on REM sleep parallels studies of sensorimotor development described above, suggesting a vital role for REM sleep in permitting developmental refinement across sensory systems. The consolidation of ODP is also reminiscent of hippocampal memory consolidation during sleep (Diekelmann and Born, 2010; Rasch and Born, 2013). These studies suggest that sleep during development is necessary for the consolidation of plastic changes induced by waking experience, which likely guide appropriate behavioral adaptations to a changing environment. Since ODP (along with other forms of developmental plasticity) occurs during a tightly restricted critical period of development, sleep disruptions early in life could have long-lasting effects on neurophysiology and behavior.

Ontogenetic changes in sleep are conserved; sleep amount and intensity are increased early in life for invertebrates, such as the fruit fly, just as they are in mammals (Jouvet-Mounier et al., 1970; Shaw et al., 2000). In *Drosophila*, 24 h of sleep deprivation following eclosion leads to long-term learning deficits, whereas adults recover from the same duration of sleep loss after one night of recovery sleep (Seugnet et al., 2011). These chronic learning impairments are likely connected with altered dopamine signaling, and can be dampened either by blocking D1 receptor activity during early life sleep loss or by elevating dopamine signaling during the days after developmental sleep deprivation (Seugnet et al., 2011). Additionally, young sleep-deprived male flies, but not mature flies, show deficits in courtship behavior as adults (Seugnet et al., 2011; Kayser et al., 2014). These courtship deficits are accompanied by decreased size of an olfactory glomerulus associated with perception of social pheromones, caused by impaired developmental growth (Kayser et al., 2014). Similarly, 1 week of early life sleep disruption impairs later social bonding in adult prairie voles (Jones et al., 2019). In this study, sleep disruption occurred during the third and fourth weeks of life, which likely falls during a critical period for maturation of GABAergic circuits that contribute to sensory integration (Gogolla et al., 2014). Notably, early life sleep deprivation in prairie voles leads to an increase in parvalbumin immunoreactivity in the primary sensory cortex, a brain region relevant to social bonding (Jones et al., 2019). Chronic changes in parvalbumin signaling could disrupt sensory processing and social behavior by altering excitatory/inhibitory balance (Yizhar et al., 2011). Together, these studies demonstrate that early life sleep is vital for developmental growth of rapidly growing brain regions across many species, and that disrupted sleep during development can result in lasting effects on adult circuitry and behavior.

While human studies have not yet revealed a mechanistic understanding of how sleep promotes neural and cognitive development, animal models indicate that sleep's role in neurodevelopment is evolutionarily ancient. Model system studies, such as those in flies and mice discussed above, have begun to examine how sleep modulates synaptic connectivity in a variety of developing sensory circuits. Further studies in these systems may reveal interventions that facilitate healthy development during insufficient sleep (Seugnet et al., 2011; Kayser et al., 2014; Jones et al., 2019).

## Learning and Memory

In a variety of species, sleep is required for several stages of memory formation and processing (Walker et al., 2002; Graves et al., 2003; McDermott et al., 2003; Ganguly-Fitzgerald et al., 2006; Seugnet et al., 2008; Krishnan et al., 2016). Indeed, sleep deprivation leads to impaired encoding (Walker et al., 2002; Yoo et al., 2007; Seugnet et al., 2008), consolidation (Graves et al., 2003; Diekelmann and Born, 2010), and retrieval (Gais et al., 2007; Lo et al., 2016; Montes-Rodríguez et al., 2019; Heckman et al., 2020) of recent associations. While even a brief nap restores memory in some assays (Seugnet et al., 2008; Ong et al., 2020), other learning and memory impairments persist after days of recovery sleep (Havekes et al., 2016; Yamazaki et al., 2020; Wu et al., 2021). While it is not clear why recovery from sleep loss varies between these conditions, studies have detected several types of longer-lasting cellular and molecular changes that persist after recovery sleep, including altered gene expression (Gaine et al., 2021), protein synthesis (Tudor et al., 2016; Lamon et al., 2021), and circuit connectivity (Weiss and Donlea, 2021). Interestingly, some types of memories seem to be more vulnerable to sleep loss than others. For example, procedural memories and memories acquired with a conscious motivation or reward benefit from sleep more than declarative or unmotivated memories (Stickgold and Walker, 2007; Diekelmann and Born, 2010). In *Drosophila*, sleep deprivation disrupts consolidation of appetitive sugar reward memories in fed flies, but in not starved flies (Chouhan et al., 2021). Together, these studies indicate that sleep deprivation likely does not have a universal effect on learning and memory, but varies based on physiological, environmental, and behavioral factors.

While the negative impacts of sleep loss on memory formation are typically detrimental, it is possible that targeted sleep disruption could be used to prevent the consolidation of maladaptive memories. Some studies, for instance, suggest that sleep deprivation could be used following trauma to degrade fear memories in patients with post-traumatic stress disorder (PTSD). Studies by Vanderheyden et al. (2015) compared sleep patterns of rats that were susceptible to developing PTSD-like symptoms after trauma to those that were resilient. While susceptible rats exhibited an increase in REM sleep in the hours following the traumatic event, resilient rats slept little during this period (Vanderheyden et al., 2015). Heightened REM sleep following trauma could lead to consolidation and reactivation of the trauma memory, preventing fear extinction, and resulting in generalization of the fear memory (Poe, 2017). Traumatic events drive activation of the mammalian locus coeruleus (LC) (Passerin et al., 2000; Naegeli et al., 2018), a collection of noradrenergic cells that promote long-term potentiation (LTP) (Izumi et al., 1992; Thomas et al., 1996; Izumi and Zorumski, 1999) and are generally quiescent during REM sleep (Foote et al., 1980). Elevated LC activity during REM sleep following a traumatic event can contribute to enhancement of recently formed emotional memories as seen in PTSD (Wassing et al., 2019). Therefore, behavioral sleep deprivation or pharmacological REM suppression following a traumatic event could lead to interventions to prevent the development of PTSD (Vanderheyden et al., 2014, 2015; Poe, 2017). Conversely, given the importance of sleep in memory consolidation (Rasch and Born, 2013) and emotional processing (Palmer and Alfano, 2017; Tempesta et al., 2018), sleep loss following a traumatic event could prevent consolidation of fear extinction memory in other conditions (Pace-Schott et al., 2015). Recent human studies have produced mixed results (Porcheret et al., 2015; Kleim et al., 2016; Cohen et al., 2017), indicating that the role for sleep in consolidating and/or maintaining traumatic memories varies with context or time elapsed since trauma. Further studies will be required to examine the therapeutic potential of sleep manipulations more clearly.

### Synaptic Plasticity and Homeostasis

Although the primary function or functions of sleep are not understood, evidence suggests a strong relationship between sleep and plasticity (Frank et al., 2001; Tononi and Cirelli, 2014). Sleep loss leads to impairments in the plastic processes of learning and memory (Diekelmann and Born, 2010; Rasch and Born, 2013). One prominent hypothesis posits that sleep's function is the renormalization of synaptic strength via downscaling of synapses that are potentiated during wake, thereby constraining excitability and restoring signal-to-noise ratios for neuronal firing (Tononi and Cirelli, 2014). Learning about the environment during waking experience requires strengthening of synapses (Clem and Barth, 2006; Gruart et al., 2006; Tye et al., 2008). According to this synaptic homeostasis hypothesis, sleep deprivation leads to cognitive deficits due to saturation of synaptic connections (Tononi and Cirelli, 2014). Evidence supporting the role of synaptic downscaling during sleep exists in a variety of species (Gilestro et al., 2009; Vyazovskiy et al., 2009; Bushey et al., 2011). At the molecular level, synaptoneurosomes from the cortex and hippocampus of adult rats display increased protein levels of GluA1-containing AMPA receptors after spontaneous and forced wake than after sleep (Vyazovskiy et al., 2008). Sleep has been found to promote synaptic downscaling in the mouse forebrain by internalizing AMPA receptors via the immediate early gene Homer1 (Diering et al., 2017). In addition, the size of the axon-spine-interface, an ultrastructural measure of synaptic strength, increases after several hours of wake compared to sleep in several mouse brain regions (Vivo et al., 2017, 2019; Spano et al., 2019). At the electrophysiological level, amplitude and/or frequency of miniature excitatory postsynaptic currents in several regions of the rodent brain increase during wake and after sleep loss, and decline following spontaneous sleep and recovery sleep (Liu et al., 2010; Bjorness et al., 2020; Khlghatyan et al., 2020). Additionally, firing rates of hippocampal and cortical neurons have been shown to increase with wake and decrease with sleep (Lubenov and Siapas, 2008; Vyazovskiy et al., 2008, 2009; Huber et al., 2013; Norimoto et al., 2018). Studies in *Drosophila* have also found increases in abundance of presynaptic and postsynaptic markers following sleep loss, consistent with the hypothesis of net potentiation during wake (Gilestro et al., 2009; Bushey et al., 2011; Huang et al., 2020; Weiss and Donlea, 2021). Additional work in the fruit fly has found that acute sleep induction is sufficient to reduce abundance of transcripts (Dissel et al., 2015) or protein (Weiss and Donlea, 2021) of synaptic components.

While evidence clearly suggests a role for sleep in synaptic downscaling in some circumstances, other studies have reported synaptic potentiation during sleep (Frank et al., 2001; Aton et al., 2013, 2014). Short periods of sleep loss decrease the number of dendritic spines in the CA1 region of the hippocampus due to increased activity of the actin-binding protein cofilin (Havekes et al., 2016). Suppressing cofilin activity in hippocampal neurons prevents spine loss and cognitive deficits following sleep deprivation, suggesting that disruption of synaptic potentiation during sleep deprivation can lead to defects in memory consolidation (Havekes et al., 2016). Similarly, sleep deprivation leads to decreased spine density in the dentate gyrus (Raven et al., 2019), and disrupts the formation of new spines following learning (Yang et al., 2014). These data indicate that, although evidence supports a general trend for synaptic downscaling during sleep, it is likely that different classes of synapses undergo different forms of plasticity during sleep or that sleep alters synaptic organization differently depending on the organism's developmental state and recent experience.

Several recent studies have sought to understand whether sleep loss differentially affects distinct classes of neurons within a single circuit or brain region. The *Drosophila* mushroom body (MB), which encodes olfactory associative memories, provides an ideal opportunity to examine the local effects of sleep loss on synapse organization. Heroic efforts have untangled the organization of the fly MB with the development of genetic drivers to label each cell type, often with single-cell resolution (Aso et al., 2014a,b) and serial reconstruction of electron micrographs have led to a detailed connectome of the MB circuitry (Li et al., 2020; Scheffer et al., 2020). These studies show that the *Drosophila* MB is an associative learning center that is divided into 15 zones defined by non-overlapping arborization of several cell types, including cholinergic Kenyon Cells (KCs), reinforcing dopaminergic neurons (DANs), and mushroom body output neurons (MBONs) which mediate behavioral valence output (Aso et al., 2014a). Associative engrams can be localized to individual zones of the MB lobes, where plasticity in the connections between odor-encoding KCs and valence-driving MBONs determines the fly's behavioral response to odorant stimuli (Aso et al., 2014b; Hige et al., 2015; Owald et al., 2015). Since sleep loss prior to training can impair acquisition/short-term memory and disrupting sleep after training prevents memory consolidation (Ganguly-Fitzgerald et al., 2006; Seugnet et al., 2008), it is likely that sleep deprivation alters either synaptic connectivity or plasticity in MB circuits. Overnight sleep deprivation selectively upscales synapses of cholinergic memory-encoding KCs, but not other cell types in the MB, including DANs or large, inhibitory interneurons (Weiss and Donlea, 2021). Further, not all types of KC output synapses are equally impacted by sleep loss; output connections from KCs to different classes of post-synaptic target neurons show wide variations in abundance following sleep loss.

**Figure 1:**
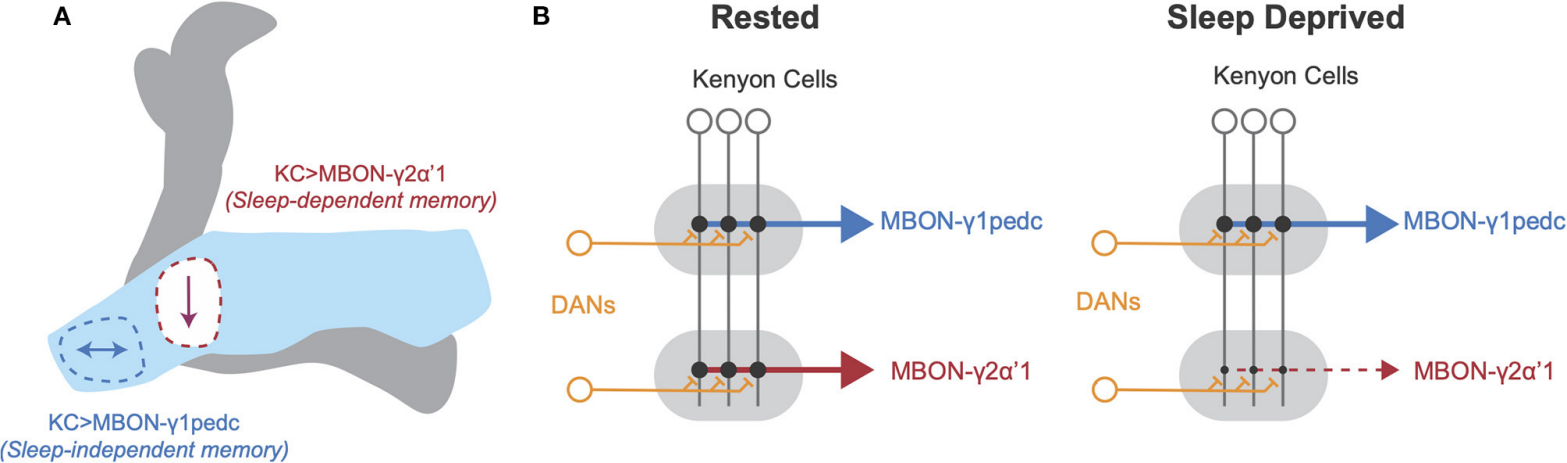
Schematic of local plasticity in *Drosophila* mushroom body after sleep loss. **(A)** Schematic illustration of *Drosophila* mushroom body. The γ lobe (light blue) contains the γ1 compartment, outlined in blue, and the γ2 compartment, outlined in red. Arrows represent changes in connectivity from Kenyon cells to MBON-γ1pedc (left, blue) and MBON-γ2α'1 (right, red). Appetitive memory encoded at KC>MBON-γ1pedc synapses is resilient to sleep loss, but appetitive memory encoded at KC>MBON-γ2α'1 synapses is impaired by sleep loss. **(B)** Schematic of connectivity between neuronal cell types in MB in rested (left) and sleep deprived brains (right). KC axons innervate tiled zones that each receive input from distinct DANs and provide input to unique MBONs. After SD, KC>MBON-γ1pedc connectivity is unchanged, but KC>MBON-γ2α'1 connectivity decreases. Based on findings from Weiss and Donlea (2021) and Chouhan et al. (2021).

Interestingly, studies by Chouhan et al. (2021), found that flies housed without food did not require sleep after appetitive conditioning to form new memories, unlike fed flies. While appetitive memory is encoded in the KC>MBON-γ2α'1 circuit in fed flies and is sensitive to sleep loss, appetitive memory is encoded in KC>MBON-γ1pedc circuitry in starved flies, and remains intact with sleep loss (Chouhan et al., 2021). Additionally, Weiss and Donlea (2021) found that sleep loss led to decreased connectivity between KCs and MBON-γ2α'1, necessary for sleep-dependent memory consolidation, while KC>MBON-γ1pedc connections, dispensable for sleep-dependent memory consolidation, were unaffected. Sleep loss could therefore disrupt consolidation of recent appetitive memories in fed flies by reducing overall connectivity between KCs and MBON-γ2α'1 (see Figure 1). Because plasticity rules can differ widely between MB sub-circuits (Hige et al., 2015), environmental conditions during learning likely influence the strength, retention, and/or decay time of a particular association. These results suggest that different zones of the MB exhibit distinct plasticity rules during sleep, likely based on learning paradigm, internal state, and other previous experience.

**Figure 2:**
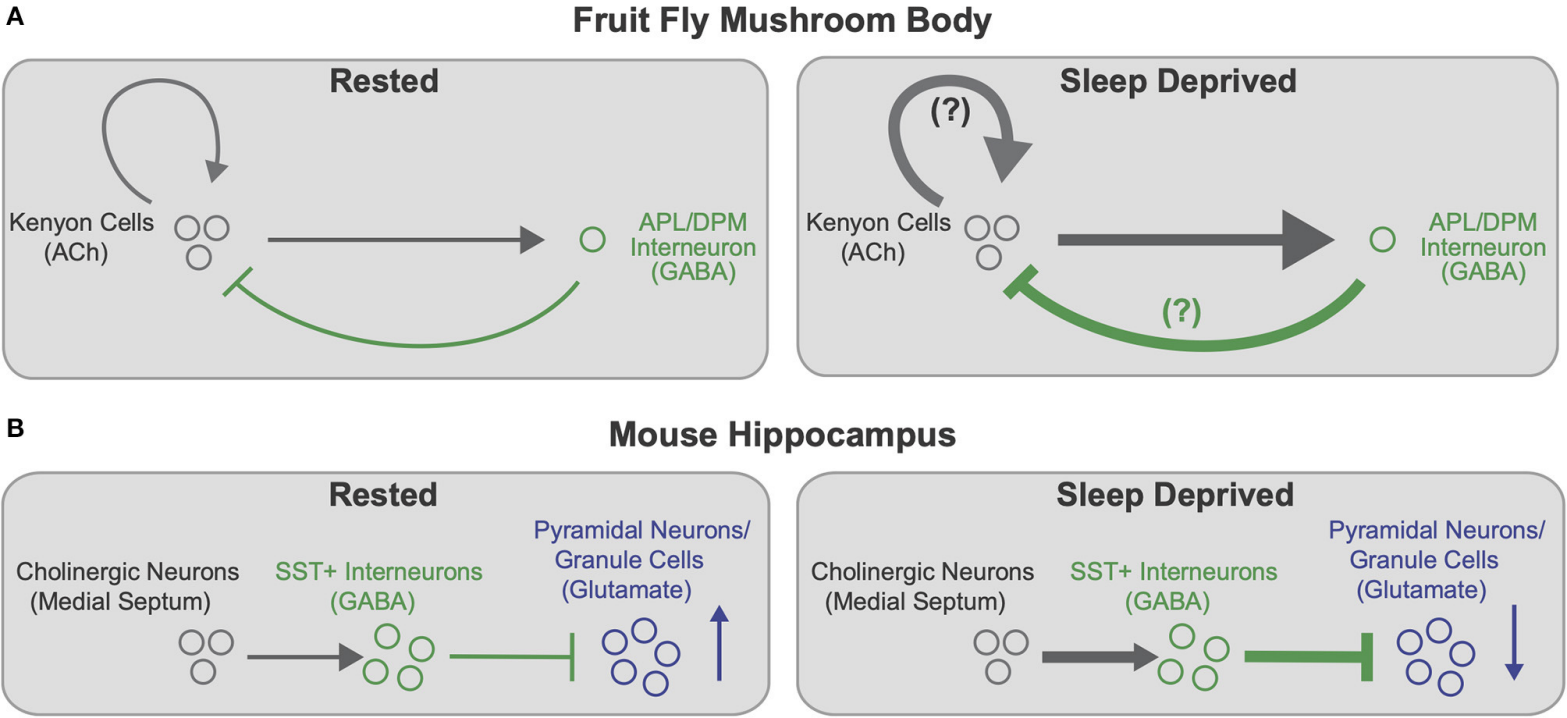
Cell type specific effects of sleep loss on memory-encoding circuits. **(A)** Schematic of connectivity between memory-encoding KCs and APL/DPM interneurons in the *Drosophila* MB in rested (left) and sleep deprived (right) flies. Cholinergic KCs activate GABAergic interneurons, which provide feedback inhibition onto KCs. KCs also synapse back onto other KCs. After SD (right), KC>APL connectivity strengthens, presumably increasing inhibition back onto KCs. KC>KC synapses may also strengthen, further contributing to increased KC>APL connectivity. Increased inhibition from APL/DPM after SD could dampen KC>KC excitation and promote recovery sleep. Based on findings from Weiss and Donlea (2021). **(B)** Schematic of hippocampal circuitry including cholinergic neurons in the medial septum to the mouse hippocampus in rested (left) and sleep deprived (right) mice. Cholinergic neurons activate GABAergic SST+ interneurons in the hippocampus, which inhibit memory-encoding pyramidal neurons/granule cells (principal neurons). After SD (right), enhanced cholinergic signaling increasingly activates SST+ interneurons, thereby heightening inhibition and reducing activity of hippocampal pyramidal neurons and granule cells. Based on findings from Delorme et al. (2021).

Supporting the idea of region and circuit specific changes in plasticity with SD, Puentes-Mestril et al. (2021) examined the effects of sleep loss on ribosome-bound transcripts for activity-dependent regulators of plasticity in excitatory pyramidal neurons and inhibitory parvalbumin-expressing interneurons. While both classes of neurons show increases in plasticity-mediating transcripts in the cortex following sleep loss, SD has little effect on abundance of these transcripts in both cell types in the hippocampus (Puentes-Mestril et al., 2021). Additional work suggests that certain cell types in the mouse hippocampus likely have privileged roles in memory consolidation during sleep (Delorme et al., 2021). Sleep deprivation leads to activation of inhibitory somatostatin-expressing (Sst+) interneurons in the hippocampus, likely due to inputs from increasingly active cholinergic neurons (Delorme et al., 2021). Both pharmacological activation of cholinergic neurons and chemogenetic activation of Sst+ cells in the dorsal hippocampus in the absence of SD leads to deficits in sleep-dependent memory consolidation (Delorme et al., 2021). Notably, both Delorme et al. (2021) and Weiss and Donlea (2021) found that sleep deprivation enhances cholinergic signaling onto GABAergic interneurons in learning/memory-related circuits, which likely increases inhibition onto memory-encoding neurons (see Figure 2). Enhanced hippocampal inhibition due to increased Sst+ activity during SD correlates with impairment of memory consolidation by disrupting LTP (Vecsey et al., 2009; Havekes et al., 2016), the reactivation of memory-encoding cells (Stefanelli et al., 2016; Clawson et al., 2021), or hippocampal oscillations (Puentes-Mestril et al., 2019). Similarly, while some inhibition from the *Drosophila* APL interneurons onto KCs is necessary to maintain spatial and temporal sparseness of odor encoding (Lei et al., 2013; Lin et al., 2014), excess inhibition would likely prevent encoding of new odor associations and reactivation of existing memory traces. Interestingly, GABAergic signaling from dorsal paired medial (DPM) and anterior paired lateral (APL) promotes sleep at night, suggesting that these interneurons may be recruited by increased KC activity during SD to promote sleep and sparsen KC representations (Haynes et al., 2015). These studies in both mice and *Drosophila* suggest that increased cholinergic signaling disrupts learning and memory after sleep deprivation, and that inhibitory drive onto memory-encoding neurons could be recruited to compensate. While these studies find complementary effects of sleep loss in the fly and mouse, these results use different approaches; Weiss and Donlea (2021) measure synaptic active zone reporters in the fly MB while Delorme et al. (2021) and Puentes-Mestril et al. (2021) quantify hippocampal transcript levels of activity-dependent immediate early genes. Additional studies will be required to directly test the relationship between connectivity changes and cell-type specific changes in activity. Ultimately, characterizing the subsets of synapses, cell types, and circuits that are most sensitive to sleep loss will help elucidate the mechanisms by which SD impairs behaviors such as learning and memory.

Sleep not only balances synaptic connectivity, but also influences neuronal firing patterns. In the rodent frontal cortex, fast spiking pyramidal cells show decreased activity during NREM sleep, while slow firing neurons increase their firing rate (Watson et al., 2016). Similar findings were observed in the mouse primary visual cortex, and these changes in firing rates were disrupted by a period of brief sleep deprivation (Clawson et al., 2018). Pyramidal neurons that are active during sleep spindles, oscillatory activity that promotes plasticity underlying memory formation (Schabus et al., 2006; Rasch and Born, 2013; Cairney et al., 2018), are increasingly active over the course of slow-wave sleep (SWS), whereas spindle-inactive pyramidal neurons show decreased activity during SWS (Niethard et al., 2021). These results indicate that sleep can increase the signal-to-noise ratio of neuronal responses by increasing the activity of sparsely firing neurons with the highest selectivity while reducing noise by decreasing activity of faster spiking, less selective neurons (Clawson et al., 2018). Interestingly, sleep during early-life ODP in mice is vital for firing rate homeostasis, indicating a potential life-long role for sleep in normalizing neuronal activity (Hengen et al., 2016; Pacheco et al., 2021).

## Resilience to Sleep Loss

### Ethological Context

While sleep contributes to many forms of experience-dependent plasticity as described above, individuals can show a wide variation in their responses to sleep loss. Sleep is homeostatically regulated across many species, but both extrinsic and intrinsic factors can influence the responses of an organism to specific sleep challenges. Food-deprived *Drosophila*, for instance, typically reduce their sleep, presumably to maximize foraging opportunities (Keene et al., 2010; Thimgan et al., 2010; Yurgel et al., 2019). While acute sleep-deprivation is typically accompanied by impaired memory and a homeostatic increase in sleep, flies that lose sleep overnight during food deprivation can retain intact memory formation and show little, if any, sleep rebound (Thimgan et al., 2010). Similarly, socially naïve male flies will also forego sleep when paired overnight with a female fly (Beckwith et al., 2017; Machado et al., 2017). This effect can be replicated by activating pheromone sensing neurons or courtship control circuits and, like starvation-induced arousal, is not followed by a sleep rebound. Similarly, the ability to temporarily offset the need for sleep has also been found in vertebrate species. Fur seals suppress REM sleep for days or weeks when foraging in seawater, accompanied by little to no REM rebound (Lyamin et al., 2018). Migratory frigate birds can reduce the time that they spend asleep by over 90% for ~10 days while continuously in flight over the Pacific Ocean compared to their sleeping patterns on land (Rattenborg et al., 2016). Similarly, Arctic male sandpipers suppress sleep for a roughly 3 week period annually while they compete for mating partners (Lesku et al., 2012). During mating season, the sun never sets in the high Arctic, allowing males to engage in unlimited visual courtship displays. Because mating success is correlated with the amount of time that male sandpipers spend awake, there is likely selective pressure for genetic factors that can allow male sandpipers to withstand prolonged sleep loss without accruing cognitive deficits or sleep drive. Constant sunlight during this period likely interacts with social and reproductive cues, enabling males to forego sleep for an extended period. Social behaviors can also drive contexts in which mammals can delay the need for sleep. Whales and dolphins, for example, can nearly fully suppress sleep for up to a month after giving birth with no recorded physiological consequences (Lyamin et al., 2005). Importantly, vertebrate sleep stages are characterized by electrophysiological signatures measured with electroencephalography (EEG) (two process model), whereas *Drosophila* sleep is defined by behavioral criteria such as quiescence and increased arousal threshold (Hendricks et al., 2000; Shaw et al., 2000). Recent work has begun to investigate whether sleep in *Drosophila* is composed of distinct stages (Yap et al., 2017; Raccuglia et al., 2019; Tainton-Heap et al., 2021), which may account for variations in plasticity and responses to sleep loss discussed above. While mechanistic studies are not feasible in many of the species mentioned here, the range of contexts in which sleep need can be temporarily offset provides exciting opportunities to understand when sleep is required for plasticity (see Table 1).

**Table 1:** Summary of experimental or ethologically-relevant conditions that reduce sleep in several species.

**Species**	**Manipulation**	**Sleep response**	**Behavioral response**	**References**
*Drosophila melanogaster*	Sleep deprivation	Decreased sleep, homeostatic rebound	Impaired learning, STM and LTM	Ganguly-Fitzgerald et al., 2006; Seugnet et al., 2008; Li et al., 2009
	Starvation	Decreased sleep, no rebound	Intact memory	Keene et al., 2010; Thimgan et al., 2010; Yurgel et al., 2019
	Stimulants	Decreased sleep	Not measured	Hendricks et al., 2000; Shaw et al., 2000; Andretic et al., 2005
	Courtship	Decreased sleep, no rebound	Not measured	Beckwith et al., 2017; Machado et al., 2017
Frigatebirds	Migration	Decreased sleep in flight, rebound on land	Not measured	Rattenborg et al., 2016
Sandpipers	Mating season	Decreased sleep	Mating success positively correlated with amount of sleep loss	Lesku et al., 2012
Cetaceans	Postpartum	Little to no sleep	Not measured	Lyamin et al., 2005, 2007
Fur seals	In seawater	Greatly reduced REM, no REM rebound	Not measured	Lyamin et al., 2018

### Intrinsic Factors

Resilience to sleep loss can also be influenced by intrinsic factors that vary between individuals. Human subjects exhibit reliable, stable responses to repeated episodes of sleep loss, suggesting that sensitivity to sleep loss can be a durable trait over time (Dennis et al., 2017; Yamazaki and Goel, 2019). Naturally occurring genetic polymorphisms coincide with an individual's response to sleep loss in flies and humans (Viola et al., 2007, 2012; Donlea et al., 2012; Satterfield et al., 2015). In two of these studies, the same genetic alleles correlated with reduced cognitive impairments and dampened homeostatic sleep pressure after prolonged waking, indicating that the identified loci could contribute to protecting neural functions during sleep loss (Viola et al., 2007; Donlea et al., 2012). Interestingly, the identified human alleles in *per3* and *tnf*α that protected individuals from the consequences of sleep loss did not predominate in the subject populations, consistent with the possibility that these alleles are accompanied with susceptibility to other physiological challenges. Brain structure can also influence sensitivity to sleep loss; variation in functional connectivity between brain regions and hippocampal structure can predict the cognitive impact of sleep loss in human subjects (Yeo et al., 2015; Saletin et al., 2016). While the neural and molecular mechanisms that connect these variations with susceptibility to sleep are not yet known, studies of model systems provide some insights into pathways that might provide protection from insufficient sleep. *Drosophila* and mouse studies have identified genetic pathways, including circadian rhythm (Mang et al., 2016; Ehlen et al., 2017), and metabolic factors (Thimgan et al., 2010, 2015), that can be manipulated to prevent rebound sleep following extended waking. It is important to note that each of these interventions can temporarily delay the accumulation of sleep debt, but it is unclear how long their protection persists and whether other consequences build as a result. Nonetheless, further examination of the external contexts and internal factors that can confer resilience to sleep loss may provide new insight into the neural functions of sleep and identify controllable interventions to facilitate rapid recovery from sleep loss.

## Conclusion

In many contexts, sleep is vital for individuals to learn and adapt their behavior to best fit their environmental conditions. Sleep facilitates brain development and circuit refinement, and early life disruptions in sleep can result in long-lasting behavioral changes. Throughout the lifespan, sleep also impacts whether new memories can be effectively acquired and consolidated. While understanding the mechanisms that contribute to sleep-dependent plasticity remain an area of intense interest, many studies have already identified molecular and synaptic connectivity changes that occur during sleep to facilitate memory formation. More clearly identifying these mechanisms and developing strategies to manipulate them could open opportunities to support cognitive processing during sleep loss. Finally, individuals exhibit varying responses to sleep loss due to intrinsic and environmental factors. Understanding the benefits and detriments of variations in sleep, as well as the biological basis for inter-individual differences, will help resolve the function(s) of sleep and elucidate how sleep patterns affect future behavior.

## Author Contributions

JW and JD wrote sections of the manuscript. All authors contributed to manuscript revision, read, and approved the submitted version.

## Funding

This work had support from NIH/NINDS R01NS105967 to JD and a Jessamine K. Hilliard UCLA Neurobiology Graduate Student Grant to JW.

## Conflict of Interest

The authors declare that the research was conducted in the absence of any commercial or financial relationships that could be construed as a potential conflict of interest.

## Publisher's Note

All claims expressed in this article are solely those of the authors and do not necessarily represent those of their affiliated organizations, or those of the publisher, the editors and the reviewers. Any product that may be evaluated in this article, or claim that may be made by its manufacturer, is not guaranteed or endorsed by the publisher.
